# Psycho-Cardiological Disease in COVID-19 Era

**DOI:** 10.31083/j.rcm2408239

**Published:** 2023-08-18

**Authors:** Peiqing Tian, Yixuan Liu, Jiayu Wang, Liyun Xing, Ping Liu

**Affiliations:** ^1^Department of Cardiology, The Second Hospital of Shandong University, 250033 Jinan, Shandong, China; ^2^School of Clinical and Basic Medicine, Shandong First Medical University, 250117 Taian, Shandong, China

**Keywords:** COVID-19, SARS-CoV-2, depression, coronary heart disease, psycho-cardiology

## Abstract

During the coronavirus disease 2019 (COVID-19) pandemic, panic and public health responses, including self-monitored quarantine and lockdown of the city, have severely impacted mental health and caused depression or anxiety in citizens. Psycho-cardiology indicates that psychological factor plays an important role in coronary heart disease (CHD). COVID-19, depression and CHD can co-exist and deleteriously affect each other, leading to worse progression and prognosis. Delays in medical consultation and treatment have become more common than before the pandemic, inducing more cardiovascular (CV) events and sequelae. COVID-19 survivors have been identified to have more psycho-cardiological symptoms compared with non-COVID-19 controls. Undoubtedly, diet alterations and sedentary lifestyles during the pandemic will cause and aggravate psycho-cardiological diseases. Some frequently used cardiovascular drugs were found to associate with changes in depression. With the advent of the post-pandemic era, although the acute damage of the Severe Acute Respiratory Syndrome Coronavirus 2 (SARS-CoV-2) is gradually declining, the psycho-cardiological diseases related to the novel coronavirus are becoming increasingly prominent. So it is an important issue for us to explore the pathogenesis, clinical manifestations and corresponding preventive measures of this aspect.

## 1. Introduction

The coronavirus disease 2019 (COVID-19) pandemic caused by the Severe Acute Respiratory Syndrome Coronavirus 2 (SARS-CoV-2) is coming to its fourth year. At the time of writing, over 615 million people were infected by the virus and more than 6.5 million people have died (https://coronavirus.jhu.edu/map.html). The extra-pulmonary impact of the disease has drawn increasing attention, and cardiovascular disease is one of the most common complications of hospitalization and death in COVID-19 patients [1].

However, COVID-19 pandemic has already been proven to impact the cardiovascular (CV) system far beyond direct damage. A series of consequences related to the pandemic, such as the panic in citizens, and public health responses, including self-monitored quarantine and lockdown of the city, have been found to increase CV risk on a wide scale, impacting both the uninfected and the survivors of COVID-19. The pandemic-related social and economic restrictions have led to economic upheaval, physical inactivity, social isolation, and mental health deterioration. All of these are recognized as CV risk factors and lead to worse outcomes [2].

Psycho-cardiology indicates that psychological factor plays an important role in coronary heart disease (CHD). A dangerous link exists among COVID-19, depression and CHD, and these elements can co-exist and deleteriously affect each other. The public health response to COVID-19, aiming at mitigating the incidence and mortality from acute infection of the pandemic, may result in a series of consequence of increasing CV risk in a much broader range, including those uninfected with SARS-CoV-2. In view of the potential harm of COVID-19 itself and its prevention and control to physical and mental health, more attention should be paid to psycho-cardiology. Thus, this review aims to make a comprehensive analysis of the interplay between COVID-19 and psycho-cardiology.

## 2. COVID-19 and Depression in the Uninfected

The lifetime risk of depression is 15–18%, meaning that nearly one in five people experience depression at some point in their lifetime [3]. The symptoms of depression can be roughly grouped into emotional, neurobiological, and cognitive symptoms [3]. Its connotation ranges from temporary discomfort to serious clinical syndromes, which can be severe, recurrent and disabling. Depression generally involves symptoms such as depressed mood, loss of interest or pleasure in activities, sleep disturbance, fatigue or impaired concentration [4]. Depression is one of the most common causes of disability in developed countries and is associated with high social and healthcare costs, including direct medical costs and reduced work productivity related to functional impairment [5].

Recent studies have identified that high prevalence rates of anxiety and depression exist among residents, in addition to patients and medical practitioners, during the COVID-19 pandemic [6, 7, 8, 9]. A meta-analysis comparing people before and after COVID-19 displayed that the prevalence of anxiety rose from 8.9% to 22.6%, while the prevalence of depression rose from 8.7% to 18.3% [10]. Several major factors of COVID-19-related anxiety are specifically listed: personal health, discrimination, social health and financial distress [11, 12]. In addition, people with current or previous COVID-19 symptoms or being closely related to the disease can take a greater psychological hit [8].

A longitudinal observational study in England suggested that the highest levels of depression and anxiety were in the early stages of lockdown, and improvements continued with lockdown-easing measures introduced [7]. Being female or young, having lower education or income levels, having pre-existing mental health conditions, and living alone or with children are all risk factors for higher levels of anxiety and depression during the lockdown [7]. On the contrary, appropriate information-seeking habits, high levels of knowledge about COVID-19, information adequacy and acceptance of public health control measures were associated with lower anxiety levels [13].

### 2.1 Depression in the Female

Self-reported stress, anxiety, depression and more severe overall psychological effects were significantly higher in women. Risk factors are more likely to intensify in women during the pandemic, including chronic environmental stress, pre-existing depression, anxiety and domestic violence [5]. Furthermore, stressors related to reproductive functioning and stages are more peculiar to women than to men during the pandemic. For example, it is reported that fertility problems, fear of accidental injury and decisional stress during pregnancy, miscarriage, additional pandemic-related worries brought by postpartum status, and intimate partner violence are aggravated to different extents [14]. As a result of school closures or family members becoming unwell, women are more likely to be responsible for additional care and household chores. During the pandemic, women are more likely to be economically disadvantaged because they have lower salaries, fewer savings and more unstable employment than men [15]. It is predicted that more severe disease prevalence among women than men will lead to greater gender differences, as women tend to be affected by the social and economic consequences of the pandemic.

### 2.2 Depression in the Young

School suspension and quarantine led to a sharp rise in the prevalence of psychological problems in students [16, 17]. A retrospective cohort study in Canada noted that by the end of 2020, the number of adolescents seeking medical care for anxiety or depression-related problems was higher than pre-pandemic levels [18]. During the pandemic, middle school students are particularly prone to anxiety, while college students are depressed [16]. Extreme fear is the most important risk factor leading to psychological distress, followed by short sleep time, being in the graduation grade, and living in areas seriously affected; sleep time mediates the influence of exposure factors on mental health [19].

### 2.3 Inappropriate Information Acquisition and Depression

Excessively frequent and constant searching for health information can lead to sleep disturbances and exacerbate mental distress such as anxiety, depression, negative perceptions of health and post-traumatic stress disorder [20]. Moreover, some information related to COVID-19, especially information obtained via social media, was significantly associated with anxiety [20]. It might be difficult for the public to distinguish true information from false. Misinformation is a significant problem in the COVID-19 pandemic. When making important decisions regarding their lives and health, ordinary people who have limited medical knowledge usually can’t tell the truth from the false. Panic purchases of food, vegetables, daily necessities, medical supplies or drugs, or even taking drugs without a prescription are related to misinformation [21]. New media consumption was also positively correlated with pandemic worries. During the pandemic, misinformation about COVID-19 spread virus-related negative effects through new media, which has caused unfounded fear and anxiety. Moreover, through new media, many netizens expressed their negative emotions, such as fear, worry, tension, and anxiety. As a result, it has caused negative emotional contagion in the online community [22].

## 3. Delays in CV Care

The COVID-19 pandemic has affected all aspects of medical services, and even impacted people uninfected with SARS-CoV-2, especially those with chronic diseases. Interaction between patients and the healthcare system is greatly disrupted, especially in areas with severe pandemics. Emergency treatment and hospitalization were delayed, deferred, or abbreviated in many patients, even for acute CV conditions [23, 24]. For instance, in the case of acute coronary syndromes (ACS) such as acute myocardial infarction (AMI), the pandemic has led to a delayed presentation to the hospital, which is associated with worse outcomes [25]. Accordingly, a two-times increase in out-of-hospital cardiac arrests has been reported during the pandemic compared to years before COVID-19 outbreak [26].

Indeed, a multicenter, international research found that symptom-to-admission times were significantly increased in patients with suspected ACS and COVID-19 who accept invasive coronary angiography [27]. Moreover, patients with any type of ACS present higher in-hospital mortality than before the pandemic, and cardiogenic shock is also more common [27]. Patients with ST-segment elevation AMI and COVID-19 were at higher risk of composite end points of in-hospital death, stroke, recurrent myocardial infarction (MI) or repeated revascularization than pre-pandemic [27]. Furthermore, these patients were more likely to develop cardiogenic shock and less likely to receive invasive angiography than pre-COVID control patients [28].

During the COVID-19 pandemic, people are understandably reluctant to be admitted to hospitals. Delays in seeking care may occur when patients fear to contact with the virus, or access to emergency medical services is limited due to reduced staffing or isolation requirements. Reasons for postponement in evaluating and treating patients after arrival in the hospital include but are not limited to nucleic acid testing, procedures for using personal protective equipment, and strict environmental disinfection [29].

As delay in medical consultation and treatment becomes more common, the incidence of CV sequelae, including cardiac remodeling, heart failure, and physical disabilities, may build up in survivors of acute CV events. Some researchers called this an “impending tsunami” [30]. Although most outpatient laboratories have resumed routine tests for cardiovascular disease, limited hospital access undoubtedly led to deferred CV risk-factor management. The duration of this phenomenon may be longer than we think or hope, because people have noticed that even three years after the SARS epidemic in 2003, the number of outpatient and inpatient visits of cardiovascular patients did not return to the level before the epidemic [31].

## 4. Crosstalk between Depression and CHD

Several psychological factors, such as depression, anxiety and type A personality have been proven to contribute to the onset as well as affect the progression and prognosis of CHD [32, 33]. CHD itself can also increase the risk of depressive symptoms and disorders, which will lead to not only direct physical consequences but also psychosocial changes. Therefore, the association between depression and CHD can be described as a downward spiral in which depression and CHD mutually reinforce each other. As the COVID-19 pandemic has caused great psychological problems, it reminds scientists and doctors to concentrate on the dangerous link between depression and CHD.

Recently, the first and largest study based on two large prospective cohorts of Chinese adults found that depression was associated with a significantly elevated risk of cardiovascular mortality, and the associations were independent of social factors, lifestyle factors, and health status [34]. Individuals with depression durations of more than two years had apparently increased risks of developing CHD, compared to those with depression of less than one year [35]. No explicit evidence revealed that depressive symptoms below threshold levels are not associated with CHD risk. The association persisted after adjusting for several known CV risk factors and attempting to eliminate the effect of reverse causality [36]. Mendelian randomization provides evidence that the relationship between raised probability of depression and increased risk of CHD is causal and genetic [37, 38].

It suggests that several established CV risk factors, such as systolic blood pressure, total cholesterol, high-density lipoprotein cholesterol (HDL-C), body mass index (BMI), diabetes, smoking, alcohol abuse, and inflammatory mediators, cannot fully explain the association between depression and CHD [36]. So, depression could be a significant predictor of incident CHD independently with other CHD risk factors [39]. In patients with established CHD, epidemiological evidence suggests a strong relationship between anxiety or depression and angina, and increased shortness of breath or chest pain symptoms are associated with depression [40].

Former studies have identified that the effect of depression on subsequent cardiac events may be mediated by abnormalities in the immune response, platelet activation and thrombosis, mitochondrial dysfunction, neuroendocrine pathways affected by altered brain and neuronal function, autonomic nervous dysfunction, life behavior and cardiometabolic risk factors [40, 41, 42]. Some new insights are being found regarding the interaction between depression and CHD. Strong associations among gut microbiota, depression, and CHD have been established. Therefore, intestinal microbiota may lead to the comorbidity of depression and CHD. Furthermore, endocrine signaling and miRNA are reported to contribute to the crosstalk between depression and CHD [41]. In view of the widespread impact on mental health of COVID-19, these could be potential targets for intervention.

## 5. Post-COVID-19 Condition

People infected with COVID-19 may have durative post-infection sequelae. The phenomenon has been given various names, such as long-term COVID-19 and long-range COVID-19. Since September 2020, it has been listed as “post-COVID-19 condition” in the ICD-10 classification and manifests itself in a variety of forms. A final consensus definition is that post-COVID-19 condition occurs in individuals with a possible or confirmed SARS-CoV-2 infection, usually three months from the onset, with symptoms that last for at least two months and cannot be explained by an alternative diagnosis [43].

### 5.1 Post-COVID-19 Condition and Psychological Symptom

Although the long-term consequences of COVID-19 remain to be studied, some COVID-19 survivors, whose physical and functional symptoms disappear after acute infection, still have problems with movement, pain or discomfort, and anxiety or depression compared with non-COVID-19 controls [44]. In a cohort study, 1733 discharged patients were investigated for the consequences of COVID-19 for six months [45]. 23% of the patients suffered from anxiety or depression, and 26% suffered from sleep difficulties. In patients with severe illness, anxiety or depression are at higher risk as a serious psychological complication.

SARS-CoV-2 can affect brain tissue by causing a cytokine storm, which is believed to cause neurological and psychiatric symptoms. The excessive and dysfunctional immune response of people infected with novel coronavirus leads to the elevation of various inflammatory cytokines. These cytokines are observed to elevate in patients with depression and are supposed to be a hypothetical mechanism different from social isolation and stressors. Due to the existence of SARS-CoV-2 in the brain, some biological alterations have been found, particularly the activation of microglia and cytokine signaling, which are alterations in psychiatric disorders in general [46]. However, the causality between cytokines induced by COVID-19 and depression needs further research.

### 5.2 Post-COVID-19 Condition and Chest Pain

Chest pain was one of the most common symptoms of the post-COVID-19 condition, with an average duration of over 40 days [47]. Another study indicates that after 60 days, 20% of cases have chest pain [48]. Although the mechanism of chest pain in post-COVID-19 conditions is still unclear, some researchers concluded that prolonged chest pain might be a consequence of coronary microvascular ischaemia based on the evidence of coronary microvascular dysfunction identified by adenosine stress CV magnetic resonance (CMR) imaging [49]. This could be a risk factor for future CHD. Besides, presence of depression is associated with increased reporting of shortness of breath and/or chest pain symptoms [40, 50].

Histopathology seems to support the role of microthrombosis in chest pain and myocardial injury [51]. Elevated cytokines, such as interleukins (IL)-1, interferon (IFN)-γ, and tumor necrosis factor (TNF)-α, could induce endothelial dysfunction, activate platelets, recruit neutrophils, and eventually trigger a hypercoagulable state, leading to myocardial injury [52].

## 6. Psycho-Cardiology in Female

Women have stronger associations between depression and CHD. They are approximately twice as likely as men to suffer from depression, and on average, somatic symptoms of depression in women are also more severe than in men, accompanied by earlier onset [40]. However, women show greater vagal activity and higher vagally-mediated heart rate variability, which are negatively associated with the risk and mortality of CHD. Possible pathophysiological mechanisms are ascribed to inflammatory processes, hormonal dysregulation, poorer health behavior and metabolic derangement modified by gender [53]. Women tend to increase their intake of unhealthy foods, decrease their physical activity and have poor sleep quality when they are anxious, leading to further increases in stress [5]. This results in an increased risk for both depression and CHD in women, and implies that gender-specific issues need to be taken into account when it comes to psycho-cardiological issues during the COVID-19 pandemic.

## 7. Psycho-Cardiology in Teenagers

Depression in childhood and adolescence is positively correlated with inflammation. It is associated with greater concurrent levels of C-reactive protein (CRP) and interleukin-6 (IL-6), as well as the increase of IL-6 in the future [54]. Conversely, elevated levels of inflammatory markers were related to future depression in teenagers. IL-6 has been identified as a potential trigger for the pathophysiology of atherosclerosis [54]. Youth depression caused by the COVID-19 pandemic may be an independent risk factor for premature CHD. Increased inflammation is also associated with more severe depression in the future, which may cause a vicious circle.

## 8. Diet Alteration in COVID-19

As a result of COVID-19, dietary and nutritional structures have been altered [55]. It was indicated that the diet adopted during the pandemic had a higher caloric intake and worse nutrition quality than the previous model of COVID-19 [56]. During the quarantine, people ate and snacked more meat, dairy, fast foods, and alcoholic beverages, but fewer vegetables, fruits and legumes [57]. Remarkably, among all populations affected by COVID-19, dietary patterns and lifestyles of overweight and obese people are notably impaired. It is frequently reported that these individuals adopt more disruptive eating behaviors, consume food without hunger, and overeat frequently [57, 58]. The expected reduction in fresh food consumption during the lockdown, accompanied by vitamin and mineral deficiencies, is associated with various CV risk factors and appears to result in higher mortality and incidence of CHD [55, 59].

Actually, adopting a Mediterranean dietary pattern, high consumption of fruits, vegetables, seafood, whole grains, nuts, and legumes, while moderate consumption of poultry, eggs, and dairy products, but only occasionally eating red meat, can reduce the burden of depression and CHD [60, 61].

## 9. Sedentary Lifestyle in Quarantine

During the COVID-19 pandemic, people who suspended work and stayed at home without exercise had poorer health indicators [62]. COVID-19 pandemic-related lockdown has led to a sedentary lifestyle among citizens of all ages. Physical exercise is one of the universal non-pharmacological interventions used to treat people with psychological disorders. Therefore, physical activity becomes especially requisite for people to maintain physiological and psychological function during the quarantine [63]. Regular physical exercises can postpone the age of the first stroke and improve long-term outcomes. This is critical on account of the higher prevalence and severity of COVID-19 in the old [64]. Although outdoor exercises are more available and various, there are still many ways to exercise at home during the quarantine, such as yoga, meditation, Tai chi, etc. [65].

Active or passive physical inactivity and positive energy balance during quarantine could induce many health consequences, including higher total body and central fat, reduced insulin sensitivity, and inflammatory status, which are the main risk factors for metabolic syndrome (MetS). An additional risk for older adults is sarcopenia combined with obesity [66].

MetS is a cluster of metabolic abnormalities, including visceral obesity, dyslipidemia, hypertension, hyperuricemia, hyperglycemia and fatty liver. It involves a series of pathophysiological, molecular biochemical, clinical and metabolic factors, directly increasing the risk of CHD, type 2 diabetes and all-cause mortality. The pathogenesis of MetS is associated with genetic and epigenetic, abnormal glucose and lipid metabolism, insulin resistance, oxidative stress, inflammation, abnormal central neurohumoral regulation and endothelial dysfunction [67]. Modern research has confirmed that a sedentary lifestyle can bring about health problems such as MetS, prothrombotic and pro-inflammatory states [66].

Even though short periods of physical inactivity gave rise to increases in TNF-α, IL-6 and CRP [68]. Adipocytes, macrophages and lymphocytes of obese individuals increase the expression levels of cytokines TNF-α and IL-6 through endocrine, autocrine and paracrine ways [67]. TNF-α acts locally on adipocytes, reducing insulin sensitivity through different mechanisms, increasing free fatty acids (FFA) levels by inducing lipolysis, and inhibiting adiponectin release [69]. It also attenuates nitric oxide-mediated vasodilation and participates in the vascular pathology of MetS, atherosclerosis and CHD [67]. IL-6 makes for insulin resistance, enhances the synthesis of acute phase proteins such as CRP and fibrinogen in the liver, promotes the expression of endothelial cell adhesion molecules, and activates the renin-angiotensin system. CRP is highly correlated with MetS and diabetes, while fibrinogen induces prothrombotic status [67, 69].

Regular physical activities activate several signaling pathways that contribute to maintaining the CV system’s steady state. Physical exercise activates the peroxisome proliferator-activated receptor-γ coactivator 1 alpha (PGC-1α) pathway, which reduces pathological myocardial remodeling, improves hypertension, reduces cardiac apoptosis and collagen accumulation, and beneficially modulates several genes related to mitochondrial biogenesis [70]. Activation of the PGC-1α pathway also helps reduce myocardial and systemic inflammation by inhibiting infiltration of macrophages, TNF-α, and inducible nitric oxide synthase, including inhibition of chemokines and cytokines in the bloodstream [71].

Physical exercise also affects angiotensin-converting enzyme 2/angiotensin-(1-7)/MAS (ACE2/Ang-(1-7)/MAS) axis, which is associated with CV pathogenesis. SARS-CoV-2 disables the positive effect of Ang 1-7 production by binding to ACE2 and entering pulmonary and other cells, inducing an imbalance between Ang II/Ang1-7 ratio and aggravating inflammatory response [72]. Contrary to pathological states, activating the ACE2/Ang-(1-7)/MAS axis by physical exercise brings about anti-inflammatory and antifibrotic effects. Physical exercise can be used as a potential therapy to promote resilience, develop an optimistic mood, and improve quality of life [73].

Exercise-based cardiac rehabilitation (CR) helps to improve the physical capacity and psychological status of psycho-cardiological patients [73]. During the COVID-19 pandemic, cardiac telerehabilitation was paid special attention to for acting as a supplement or substitution to traditional centre-based CR [74]. Telerehabilitation was identified to improve lipid particles recently, and it may create a combined effect of multiple behavior modifications and risk reduction for psycho-cardiological patients [75].

## 10. Psycho-Cardiology in Special Population

Hypertension and diabetes mellitus are all the most common co-morbidities and causes of death in patients with COVID-19 infection [76], and they are also known as risk factors for CHD. So patients with hypertension or diabetes mellitus should be brought to the forefront.

### 10.1 Hypertensive Patients

In a prospective study, compared to O blood group in hypertensive patients with COVID-19 infection, non-O patients had significantly higher values of pro-thrombotic indexes (activated pro-thrombin time, D-dimer, Von Willebrand factor and Factor VIII), higher rate of cardiac injury (13.9% vs. 29.3%) and higher mortality (8.3% vs. 19.6%) [77]. Hypertension could exacerbate pro-thrombotic status, over-inflammation and endothelial dysfunction in COVID-19 patients, resulting in an increased risk of worse prognoses as cardiac injury and death [77].

The use of anti-hypertensive drugs was once in controversy. In fact, keeping taking ACE inhibitors/angiotensin receptor blockers (ARBs) to control hypertension along with tailored anti-inflammatory and immune therapies could improve clinical outcomes, and prevent worse prognosis in hypertensive patients with COVID-19 [78]. ACE inhibitors and ARBs could mediate COVID-19 protection by anti-inflammatory, anti-fibrotic, and anti-thrombotic effects and improvement of lung function via upregulating ACE2 activity [78, 79].

### 10.2 Diabetes Patients

During COVID-19 infection, diabetes patients show high prevalence, severity of disease and mortality. COVID-19 pneumonia could cause thromboembolic events and reduction of lung functionality, especially in patients with diabetes [80]. These events are manifestations of micro vascular endothelial dysfunction and damage, which are also thought to be risk factors for CHD. ACE2 expression (total and glycosylated forms) was upregulated in patients with worse glycemic control, resulting in myocardial injury under COVID-19 infection [81]. In fact, early glycaemic control was indicated to be a suitable therapeutic option to improve prognosis in hospitalized hyper-glycaemic COVID-19 patients with or without a previous diabetes diagnosis [82]. Besides, hyper-glycaemia at the time of vaccination worsened the immunological response, and increased incidence of SARS-CoV-2 breakthrough infections [83, 84], and thus influenced the efficacy of vaccination.

## 11. CV Drugs and Depression

During the COVID-19 pandemic, particular attention should be paid to medication for psycho-cardiological disease patients. A study based on data of 5.4 million people in Denmark investigated whether CV drugs were associated with changes in depression. Continued use of angiotensin agents, calcium channel blockers (CCB), and β-blockers were associated with decreased rates of depression, whereas diuretics were not. Reduced risks of depression were found in nine drugs, including two angiotensin agents: enalapril and ramipril; three calcium antagonists: amlodipine, verapamil, and verapamil combinations; and four β-blockers: propranolol, atenolol, bisoprolol, and carvedilol. None of the antihypertensive drugs was found to increase the risk of depression [85]. Previous studies have confirmed that statins help to reduce the risk of depression in people with CHD [86, 87]. The anti-depression effect of aspirin is still controversial, and nitrate drugs were not significantly associated with depression [88]. However, a recent meta-analysis suggests that CCB, diuretics and nitrate are associated with higher risks of depression in patients with CHD and heart failure [89]. This could be a research priority, given that CV drugs’ effects on depression remain controversial.

## 12. Conclusions

The public health responses to the COVID-19 pandemic have impacted citizens on a broad scale, especially in the psychological and CV aspects. Females and the young are susceptible populations of psycho-cardiological diseases induced by COVID-19. Patients with hypertension or diabetes mellitus should especially be brought to the forefront during the pandemic. Inappropriate information acquisition, delays in CV care, post-COVID-19 condition, diet alteration and sedentary lifestyle in quarantine, however, would cause or aggravate psycho-cardiological diseases. With entering the post-pandemic era, the acute effects of SARS-CoV-2 gradually decline, so more attention should be paid to its chronic consequence, particularly psycho-cardiology. Mediterranean dietary pattern and exercise-based cardiac rehabilitation are essential for preventing and treating psycho-cardiological diseases.

## Abbreviations

ACS, Acute coronary syndromes; AMI, Acute myocardial infarction; BMI, Body mass index; CHD, Coronary heart disease; COVID-19, Coronavirus disease 2019; CRP, C-reactive protein; CV, Cardiovascular; HDL-C, High-density lipoprotein cholesterol; IFN, interferon; IL, Interleukins; MetS, Metabolic syndrome; SARS-CoV-2, Severe Acute Respiratory Syndrome Coronavirus-2; TNF, tumor necrosis factor.
